# Angular Velocity Affects Trunk Muscle Strength and EMG Activation during Isokinetic Axial Rotation

**DOI:** 10.1155/2014/623191

**Published:** 2014-04-08

**Authors:** Jian-Zhong Fan, Xia Liu, Guo-Xin Ni

**Affiliations:** ^1^Department of Rehabilitation Medicine, Nanfang Hospital, Southern Medical University, Guangzhou 510515, China; ^2^Department of Rehabilitation Medicine, Binzhou Medical University Hospital, Binzhou 256603, China; ^3^Department of Orthopaedics and Traumatology, Nanfang Hospital, Southern Medical University, Guangzhou 510515, China

## Abstract

*Objective*. To evaluate trunk muscle strength and EMG activation during isokinetic axial rotation at different angular velocities. *Method*. Twenty-four healthy young men performed isokinetic axial rotation in right and left directions at 30, 60, and 120 degrees per second angular velocity. Simultaneously, surface EMG was recorded on external oblique (EO), internal oblique (IO), and latissimus dorsi (LD) bilaterally. *Results*. In each direction, with the increase of angular velocity, peak torque decreased, whereas peak power increased. During isokinetic axial rotation, contralateral EO as well as ipsilateral IO and LD acted as primary agonists, whereas, ipsilateral EO as well as contralateral IO and LD acted as primary antagonistic muscles. For each primary agonist, the root mean square values decreased with the increase of angular velocity. Antagonist coactiviation was observed at each velocity; however, it appears to be higher with the increase of angular velocity. *Conclusion*. Our results suggest that velocity of rotation has great impact on the axial rotation torque and EMG activity. An inverse relationship of angular velocity was suggested with the axial rotation torque as well as root mean square value of individual trunk muscle. In addition, higher velocity is associated with higher coactivation of antagonist, leading to a decrease in torque with the increase of velocity.

## 1. Introduction

Low back pain (LBP) is an extremely common problem affecting about two-thirds of people at some time in their life and listed as the second symptom-related reason for visiting a physician [1, 2]. The etiology of LBP is very complex and could be attributed to different causes. Among them, biomechanics played a major role in spinal pathology and pain. For one thing, trunk muscles are the crucial contributor to the spine stability [3, 4]. It is believed that trunk muscle weakness is an important risk factor for this disorder [5]. Additionally, the abnormal patterns of muscle activity could affect the biomechanics of spinal movements and result in mechanically induced pain [6–8].

The biomechanical characteristics of spinal movement have been investigated using isometric or isokinetic testing with/without EMG measurement but mainly during trunk flexion-extension [9–13]. Due to the complicated nature of axial rotation or limited to technique, relatively much less attention has been paid to axial rotation exertion. Actually, it was reported that trunk torque generating capacity in isometric rotation-flexion was more affected by rotation than flexion [14]. What is more, results from an epidemiological study showed that trunk rotation, a common activity in daily life, has been associated with over 60% of the reported lower-back injuries [15]. As such, more information is needed to understand the trunk rotatory muscle function.

The static function of trunk muscles has been evaluated during isometric axial rotation by a number of research groups [16–21]. EMG measurement has been extensively used to assess the pattern and magnitude of trunk muscles in maximal isometric and graded isometric axial rotational contractions [16, 18, 22] and identify their functional roles as a result [20, 23]. In comparison with isometric measurement, isokinetic measurement has better reliability and precision [24–26] and has been increasingly used for dynamic strength measurement to understand the mechanical profile of skeletal muscle, including trunk muscles [27, 28]. Specifically, a number of studies were aimed at the trunk rotator muscle performances using this measurement in various kinds of subjects [29, 30]. Nevertheless, few used additional EMG measurement to estimate individual trunk muscle activities [31].

During various activities, rotation of the trunk is performed at different velocities. The muscular torque during isokinetic testing was reported to decrease with increasing angular velocity of movement [27–30]. However, little is known as to how trunk muscles activate at different velocities. Complex linking between the torque, velocity, and EMG was ever suggested, which may be effective in maintaining the safety of a joint by keeping the resultant from increasing beyond the margin of safety [32]. So far, only one study has been found to evaluate the activation of trunk muscles in an isokinetic model at different velocities [31]. To better understand the relationships between torque, velocity, and EMG, this study was performed to investigate the biomechanical characteristics of trunk rotator muscles and EMGs of three pairs of trunk muscles in isokinetic modes at three angular velocities (30°/s, 60°/s, and 120°/s).

## 2. Methods

### 2.1. Subjects

Twenty-four healthy male volunteers without any history of back pain participated in this study. All the subjects were right-handed collegiate male from Southern Medical University with mean age, height, and weight of 26.5  years, 173.6 cm, and 66.7 kg, respectively. Written informed consent was obtained from all the subjects before participation. This study was approved by the Institutional Review Board, Nanfang Hospital, Southern Medical University.

### 2.2. Experimental Procedure

**Figure 1:**
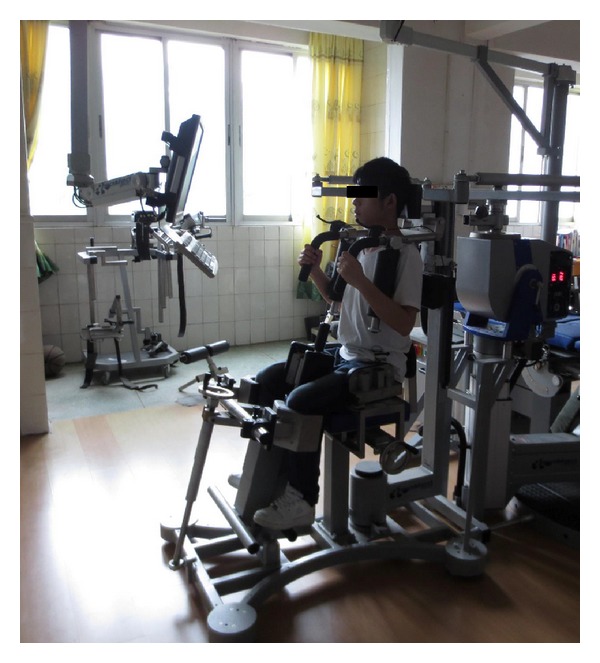
Subject was seated in the Isomed 2000 Torso Rotation isokinetic dynamometer for trunk rotation testing.

The subjects were placed in the Isomed 2000 Torso Rotation isokinetic dynamometer (Isomed 2000, D&R, Hemau, Germany) in a seated position with 90° of hip and knee flexion (Figure 1). The reliability of the instrumentation has been evaluated previously [24]. The subjects' feet were secured in straps to a platform that could be adjusted to produce a consistent hip and knee position. A 90° arc of total rotation was set using range of motion stops (45° of right and left rotation). In the current study, three angular velocities (30°/s, 60°/s, and 120°/s) were chosen for isokinetic testing. Prior to formal testing, all subjects attended the familiarization session, so that they may gain some knowledge of the equipment and testing procedure, thus minimizing the learning effect. Afterwards, each subject underwent 5 maximal repetitions at 30°/s, 10 maximal repetitions at 60°/s, and 15 maximal repetitions at 120°/s. A 60 s rest period was used between sets. During the testing protocol, consistent verbal commands and visual feedback were given to exert maximal effort throughout the whole range of motion [33]. The whole procedure was operated by a well-trained examiner.

The surface EMG activities of three pairs of trunk muscles (latissimus dorsi (LD), external oblique (EO), and internal oblique (IO)) were recorded using a portable EMG system (ME6000; Mega Electronics Ltd., Finland). Before the electrode was placed, the surrounding area was shaved using a disposable razor and disinfected using alcohol-soaked cotton wool. Surface Ag/AgCl electrodes (Shanghai Shenfeng Medical & Health Articles Co., Ltd., Shanghai, China) were chosen with an interelectrode distance of 30 mm. The positions to place surface electrodes were determined by palpation of each muscle belly. Electrodes for LD were placed at T12 level and along a line connecting the most superior point of the posterior axillary fold and the S2 spinous process. The T12 level was selected so as to avoid the pressure of the thoracic pad on the electrode. For EO, electrodes were placed just below the rib cage and along a line connecting the most inferior point of the costal margin and the contralateral pubic tubercle [34]. Electrodes for IO were placed 1 cm medial to the anterior superior iliac spine (ASIS) and beneath a line joining both ASISs [35].

### 2.3. Data Analysis

**Figure 2:**
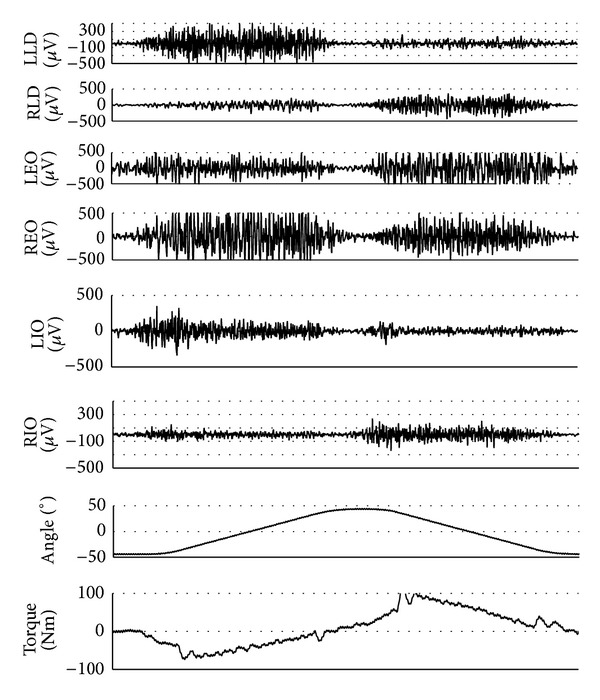
Individual recording of axial rotation torque and raw EMG signals of three pairs of muscles with its corresponding trunk rotation angle in an isokinetic model. LLD: left latissimus dorsi; RLD: right latissimus dorsi; LEO: left external oblique; REO: right external oblique; LIO: left internal oblique; RIO: right internal oblique.

All recorded data were analog-to-digital converted using 1 kHz sampling rate and stored in a personal computer. EMG signals were sampled together with data from the dynamometer using Power and Signal Software (Isolation Unit, ME4ISO, Mega Electronics Ltd., Kuopio, Finland). Parameters measured by the dynamometer included peak torque, peak torque relative to body weight, single maximal repetition work, and power. For each muscle at each velocity, the amplified raw EMG signals within 200 ms epoch (i.e., 100 ms before and after the time to peak torque) were converted to the root mean square (RMS) values to quantify the amplitude of EMG signals (Figure 2).

### 2.4. Statistics

All statistical analyses were conducted using SPSS software (SPSS Inc., Chicago, IL). One-way ANOVA was used to compare trunk rotation torque, work, power, and angle at peak torque (APT), namely, the joint angle at which the PT was achieved, among different velocities. RMS values were compared using two-way ANOVA with repeated measures (muscle × angular velocity). Bonferroni's post hoc analysis was conducted if the ANOVA showed statistically significant main effects or interaction effects. All data were presented as means ± standard deviations. Statistical significance was set as *P* < 0.05.

## 3. Results

**Figure 3:**
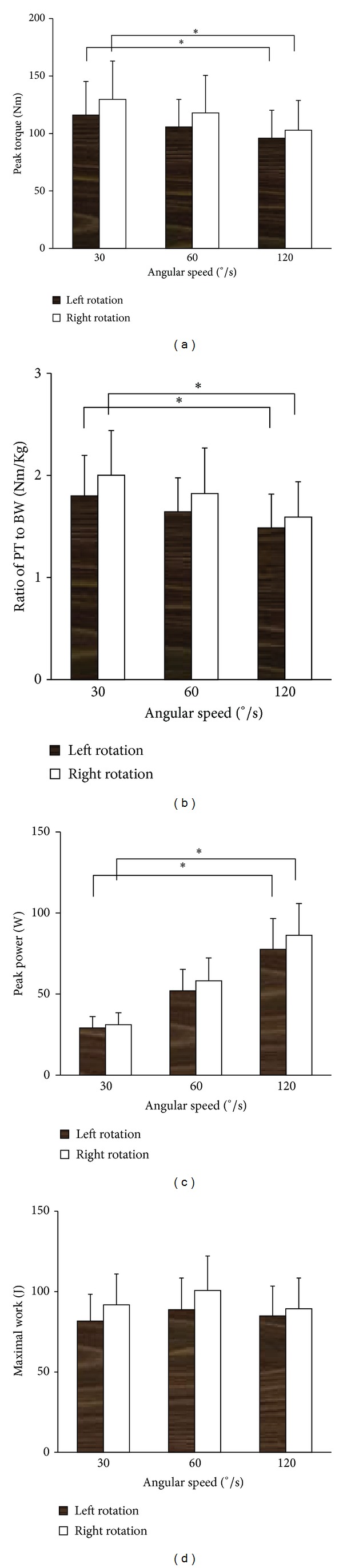
Parameters measured by the dynamometer at three different velocities, including peak torque (a), ratio of peak torque to body weight (b), single maximal repetition work (c), and power (d). Both PT and PT/BW decreased with the increase of angular velocity. Conversely, the peak power increased with the increase of angular velocity in each direction. As for these parameters, significant difference was found between 30°/s and 120°/s. No significant difference was found among different angular velocities in single maximal work. *P < 0.05, compared with that at 120°/s.

**Figure 4:**
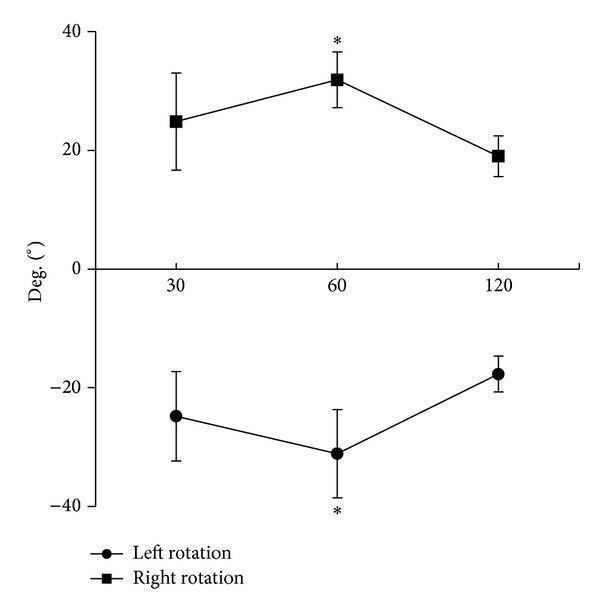
Angle at peak torque (APT) at three velocities in both directions of trunk rotation. In each direction, the highest APT was found at 60°/s, which was significantly higher than that at 120°/s. *P < 0.05, compared with that at 120°/s.

**Figure 5:**
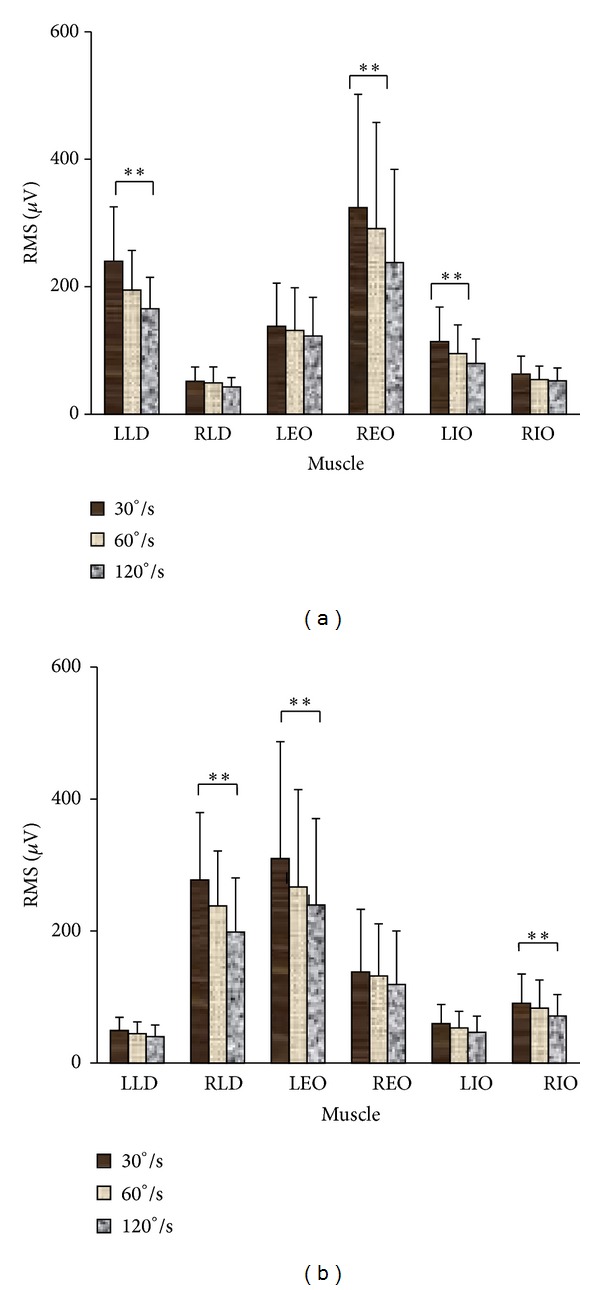
The RMS value of each muscle at different velocities in both directions ((a) left rotation; (b) right rotation). Among three primary agonists in each direction, external oblique exhibited the highest RMS value during isokinetic axial rotation. For each primary agonist, the RMS value decreased with the increase of angular velocity. **P < 0.01, compared between that at 30°/s and 120°/s.

**Figure 6:**
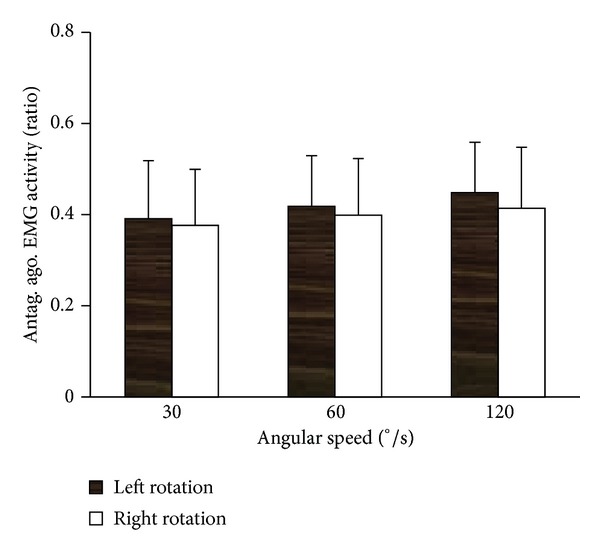
The RMS ratios of three antagonists to three agonists at three velocities in both directions. Regardless of the angular velocity, coactivation phenomenon was observed. The RMS ratio increased with the increase of velocity in each direction, suggesting that antagonist exhibited relatively higher activation during fast movement.

During and after isokinetic trunk rotation, no symptoms of overuse injuries including low back pain and fatigue were reported. The isokinetic trunk muscle rotator performance was shown in Figures 3–6. As for peak torque (PT), the left-to-right rotation ratio was 0.91 at 30°/s, 0.92 at 60°/s, and 0.94 at 120°/s, respectively. In each direction, PT and PT/BW (PT relative to body weight) decreased with the increase of angular velocity, and there were significant differences between 30°/s and 120°/s (Figures 3(a) and 3(b)). Conversely, the power increased with the increase of angular velocity in each direction, with significant difference existing between 30°/s and 120°/s (Figure 3(c)). No significant difference was found among different angular velocities in single maximal work (Figure 3(d)).

In the present study, APTs were calculated at three velocities in both directions of trunk rotation (Figure 4). In each direction, the highest APT was found at 60°/s, which was significantly higher than that at 120°/s. However, no side differences were found at each angular velocity.

Figure 2 illustrated the EMG activity of three pairs of trunk muscles at three angular velocities in both directions. It suggested that, during isokinetic axial rotation, contralateral EO and ipsilateral LD and IO acted as primary agonists, whereas ipsilateral EO and contralateral LD and IO acted as primary antagonistic muscles. The RMS value of each muscle was shown in Figure 5 at different velocities in both directions. Among three primary agonists in each direction, external oblique exhibited the highest RMS value during isokinetic axial rotation. For each primary agonist, the RMS value decreased with the increase of angular velocity, and statistical difference was found between that at 30°/s and 120°/s.

In addition to RMS value of individual muscle in each direction, the RMS ratios of three antagonists to three agonists were also calculated at each velocity in both directions (Figure 6). Regardless of the angular velocity, coactivation phenomenon was observed. However, the RMS ratio increased with the increase of velocity in each direction, without statistical significance though, suggesting that antagonist exhibited relatively higher activation during fast movement. At each velocity, no significant difference was found between left and right rotation.

## 4. Discussion

In the current study, the effect of angular velocity was investigated on the biomechanical characteristics of trunk rotator muscles and EMGs activities of 3 pairs of trunk muscles in an isokinetic mode. Our findings suggested that angular velocity has great impact on isokinetic trunk muscle rotator performance and EMG activities of individual trunk muscle. An inverse relationship of angular velocity was suggested with the axial rotation torque as well as RMS value of individual trunk muscle. What is more, during isokinetic axial rotation, higher coactivation of antagonist was found at higher velocity, contributing to a decrease in torque with the increase of velocity.

The axial rotation torque of young males was assessed at three angular velocities, and an inverse relationship was suggested between the axial rotation torque and the velocity of rotation. Similar results were ever reported in healthy subjects by Lindsay and Horton [29] and Kumar et al. [31] using different angular velocities. Such relationship also existed in elite male golfers and sedentary controls with and without low back pain [27, 29]. Nevertheless, apparent decrease in the peak torque was not shown in hemiplegic patients with the increase of angular velocity, probably due to insufficient use of high threshold motor units induced by the hemispheric lesion [36]. Contrary to peak torque, peak power rose as angular velocity increases. Peak power is defined as the product of moment and angular velocity. It is therefore suggested that, as angular velocity increases, the magnitude of the torque drop is not sufficient to offset the effect of speed increase, thus producing a rise in power. In isokinetics, work is defined as the area under the torque versus angular displacement (time) curve (work = torque × distance). No significant difference in muscular work was found in our study among different velocities, suggesting that the duration of peak torque increases along with the PT drop as angular velocity increases.

APT reflects the joint position at which that trunk muscle exhibits maximal exertion. Some investigations were previously reported on knee and shoulder, to provide information about the mechanical properties of the contracting muscles [37, 38]. Our study is the first assessing APT on trunk rotation. Our results showed that APT was velocity dependent, and in both directions, the APT at 60°/s was bigger than that at 30°/s and 120°/s, respectively. Although the exact reason remains unknown, this finding should be of clinical relevance. A muscle shows the maximal tension only when its length is in the appropriate level. Placing the muscle in a shorter or longer length from its optimal length by extending or flexing the joint, respectively, will lower force output. In this regard, the angular position is important in the assessment and rehabilitation of trunk rotator muscle function.

EMG activities were also examined in the current study on three pairs of trunk muscles during isokinetic axial rotation, and the results showed that, for each muscle, the RMS value decreased as the velocity increases. Our findings were consistent with Kumar et al.'s study [31], in which EMG activities were compared among 10, 20, and 40°/s. Through intermuscle comparison, it was demonstrated that, during isokinetic axial rotation, the contralateral external oblique, ipsilateral latissimus dorsi, and internal obliques acted as primary agonists, whereas ipsilateral external oblique, contralateral latissimus dorsi, and internal obliques acted as primary antagonistic muscles. Similar functional roles were demonstrated of external oblique and internal oblique abdominal muscles during isometric axial rotation [20, 21]. However, less attention has been paid to latissimus dorsi during axial rotation. This muscle is capable of producing rotational torque [39, 40] as well as providing stability to the lumbar spine [39]. Therefore, it is not surprising for its high activation during axial rotation observed in the current study. Marras and Granata [39] suggested that part of the activity in the latissimus dorsi was to counteract the ancillary torque produced by rectus abdominis.

Antagonist coactivation is quite important for the stiffness and stability of a joint, and higher coactivation level is more likely during fast than slow movements [41]. Such phenomenon was reported by a number of research groups, mainly on knee flexors (hamstrings) [42, 43]. Our study is the first reporting coactivation of antagonist during axial trunk rotation. The RMS ratio of antagonistic to agonist muscle activity increased with the increase of velocity, suggesting that antagonist exhibited relatively higher activation at higher velocity. As some of the antagonist activations are most likely needed to equilibrate the moment produced by the trunk agonist [44–46], it is believed that a large component of the increased antagonist activation with increasing velocity was related to neuromuscular control and the attempt to minimize kinematic variability. In addition, with the increase of angular velocity, relatively higher coactivation level in antagonist would lead to the decrease in peak torque, reported in the current and many other studies [41, 47].

The limited number of subjects in this study, the nature of the sample (volunteers rather than randomly selected subjects), and characteristic of the sample (only collegiate male recruited and lifestyles not considered) required that caution be exercised in using the results as “normative” torque- and EMG-velocity relationships. However, the data do indicate such relationships for healthy young men of sedentary to moderately active lifestyle. These data can serve as a baseline for comparing subject data and for collecting further information.

## 5. Conclusion

Our study suggested an inverse relationship of angular velocity with the axial rotation torque as well as RMS value of individual trunk muscle, indicating that velocity of rotation has great impact on the axial rotation torque and EMG activity. Our study also found that higher velocity is associated with higher coactivation of antagonist, which may explain the decrease in torque with the increase of velocity. More investigations are needed to further understand the linking between torque, EMG, and velocity, as well as its implication in the assessment and rehabilitation training of trunk rotation muscles. The major implications of our findings are twofold. First, since torque- and EMG-velocity relationships are specific to a given individual, assessing these parameters could help clinicians to target and adjust their training programs and exercises to the patient's abilities, so as to design personalized training and rehabilitation program. The second implication relates to rotational jobs, which can be designed to control rotational back injuries, based on the quantitative data presented.
